# The economic impact of hypercholesterolemia and mixed dyslipidemia: A systematic review of cost of illness studies

**DOI:** 10.1371/journal.pone.0254631

**Published:** 2021-07-12

**Authors:** Pietro Ferrara, Danilo Di Laura, Paolo A. Cortesi, Lorenzo G. Mantovani

**Affiliations:** 1 Center for Public Health Research, University of Milano–Bicocca, Monza, Italy; 2 Value-based Healthcare Unit, IRCCS MultiMedica, Sesto San Giovanni, Italy; Icahn School of Medicine at Mount Sinai, UNITED STATES

## Abstract

Hypercholesterolemia is a clinically relevant condition with an ascertained role in atherogenesis. In particular, its presence directly correlates to the risk of atherosclerotic cardiovascular disease (ASCVD). As known, cardiovascular diseases pose a significant economic burden worldwide; however, a clear picture of the economic impact of ASCVD secondary to hypercholesterolemia is lacking. This study aiming at conducting a systematic review of the current literature to assess the economic impact of familial hypercholesterolemia (FH), non-familial hypercholesterolemia (non-FH) or mixed dyslipidemia. A literature search was performed in Medline/PubMed and Embase database up to September 1^st^, 2020, exploring evidence published from 2010. The literature review was conducted in accordance with PRISMA guidelines. To be included the studies must be conducted on people who have been diagnosed with familial hypercholesterolemia, non-familial hypercholesterolemia or mixed dyslipidemia, and report data/information on costs attributable to these conditions and their sequelae. A total of 1260 studies were retrieved. After reading the titles and abstract, 103 studies were selected for full reading and eight met the criteria for inclusion. All but one studies were published in the American continent, with the majority conducted in US. An observational design with a prevalence approach were used and all estimated the economic burden of CVD. Direct cost estimates as annual average health expenditure on all population, ranging from $17 to $259 million. Few studies assessing the economic impact of hypercholesterolemia are available in the literature and new researches are needed to provide a more updated and reliable picture. Despite this scarceness of evidence, this review adds important data for future discussion on the knowledge of the economic impact of hypercholesterolemia and costs of care associated to this condition, with important implication for public health researches and novel therapies implementation.

## Introduction

Hypercholesterolemia is defined as the presence of high level of plasmatic low-density lipoprotein–cholesterol (LDL-C), with a clinically significant role in the developing of atherogenesis, and being directly correlated to the risk of atherosclerotic cardiovascular disease (ASCVD) [1]. In mixed dyslipidemia, hypercholesterolemia is associated with elevated triglyceride (TG) levels and low levels of high-density lipoprotein–cholesterol (HDL-C), contributing significantly to the overall cardiovascular risk [2].

Cardiovascular diseases (CVD) are estimated to cost more than $863 billion worldwide, posing a significant economic burden that is expected to reach $1,044 billion in 2030 [3]. In this context, the increasing prevalence of hypercholesterolemia and dyslipidemia leads to a massive cost allocation for the healthcare systems in industrialized countries over the next years [3]. Costs items contributing to this financial impact are associated to the management of the health consequences and disease-related events, such as ASCVD, and to cost-minimizing (compared with proved equivalent therapeutics) preventive interventions (e.g., screening and pharmacological treatments), which are both are associated to significant healthcare resources consumptions (e.g. hospitalization, diagnostic procedures and revascularization, medical follow-up, and chronic use of several medications) [4,5].

Health expenditure attributable to ASCVD has been the subject of some national research, but a clear picture of the studies on the economic impact of ASCVD secondary to hypercholesterolemia and mixed dyslipidemia is lacking, with available studies conducted at country level with small patient groups or focusing on specific economic aspects associated to the condition [4–6].

Due to the considerable cost impact of hypercholesterolemia and mixed dyslipidemia, it is necessary to have a better understanding of the economic burden associated to these conditions summarizing, in a systematic way, the evidence available on the cost-of-illness. The objective of this research is therefore to summarize the current evidence that assess the economic impact of familial hypercholesterolemia, non-familial hypercholesterolemia or mixed dyslipidemia, by systematically reviewing existing literature.

## Materials and methods

### Target population and outcomes

The target population of this review was the people who have been diagnosed with familial hypercholesterolemia, non-familial hypercholesterolemia or mixed dyslipidemia. For the purpose of this study, hypercholesterolemia was intended as the rise in levels of plasmatic LDL-C; familial hypercholesterolemia as inherited autosomal dominant genetic lipid disorder that causes of hypercholesterolemia; mixed dyslipidemia as a condition where hypercholesterolemia is associated with high TG and low HDL-C levels [1,2].

No restriction on the age of participants was applied. The main outcome measured was the overall costs associated to these conditions, stratified as direct and indirect costs. The former referred to health care expenditure and included costs due to treatment, complications and interventions of familial hypercholesterolemia or mixed dyslipidemia. Indirect costs were considered as the value of income lost due to disease and complications in the working-age population.

### Literature search

Medline/PubMed and Embase databases were systematically queried for relevant literature. Searches were performed up to September 1^st^, 2020, exploring evidence published from 2010 onwards to focus on more relevant data applicable to the current scenario of the management and impact of hypercholesterolemia and mixed dyslipidemia. No restriction on publication status was applied. The databases were surfed with the terms (both as MeSH term and free-text keywords) “hyperlipoproteinemia”, “hypercholesterolemia”, “dyslipidemia” and one among “cost of illness”, “cost”, “cost analysis”, “economic”, “economic burden”, included in the search strategy described in S1 Appendix (Supplementary material). The lists of references of the included studies were manually screened in order to include relevant papers not retrieved previously. Efforts to incorporate all potentially relevant reports also included hand-searching of grey literature. According to investigators’ language skills, reports in English, Spanish, Italian, Portuguese, and French were considered for inclusion in this analysis. The literature revision was conducted in accordance with the Preferred Reporting Items for Systematic Reviews and Meta-Analyses (PRISMA) guidelines 2020 [7].

### Study selection

Selection criteria for article screening and inclusion in the review were: (1) primary peer-reviewed articles accessible in full-text; (2) including patients diagnosed with familial hypercholesterolemia, non-familial hypercholesterolemia or mixed dyslipidemia; (3) reporting data/information on costs attributable to the disease. Records that met the following criteria were excluded: (1) studies without defined outcome measures; (2) not including cost-of-illness data; (3) published as review, congress abstract, editorial, or letter to editor; (4) or published in other languages than those listed above.

### Data extraction, data synthesis, and quality assessment

The assessment of titles, abstracts, and full texts for relevant articles was independently conducted by three reviewers (PF, DDL, PAC). Possible disagreements were resolved by group discussion, until reaching of consensus. Data of each study were tabulated in a pre-defined form. For each article, the following baseline characteristics were extracted: first author’s last name, country and year of publication, study design, data source and year of data collection, study population and subgroups, clinical outcomes, characteristics and description of costs attributable to illness (cost data and items, estimation methods, year and currency). Additionally, two reviewers (PF and PAC) critically assessed the quality of the economic evaluations retrieved by the systematic literature review, according to the Consensus Health Economic Criteria (CHEC) list [8]. Disparities in cost items and in methods of calculation of the economic impact of the diseases across the include studies affected the possibility of pooling results into meta-analysis and findings were therefore presented as narrative synthesis. However, to allow comparability among the results from each study, costs were firstly transformed using Purchasing Power Parities (PPP) for gross domestic product to US dollar (USD) [9], and then inflated by 1 percent annually to reach a common end value for the year 2020, as suggested in another similar research [10].

## Results

**Fig 1:**
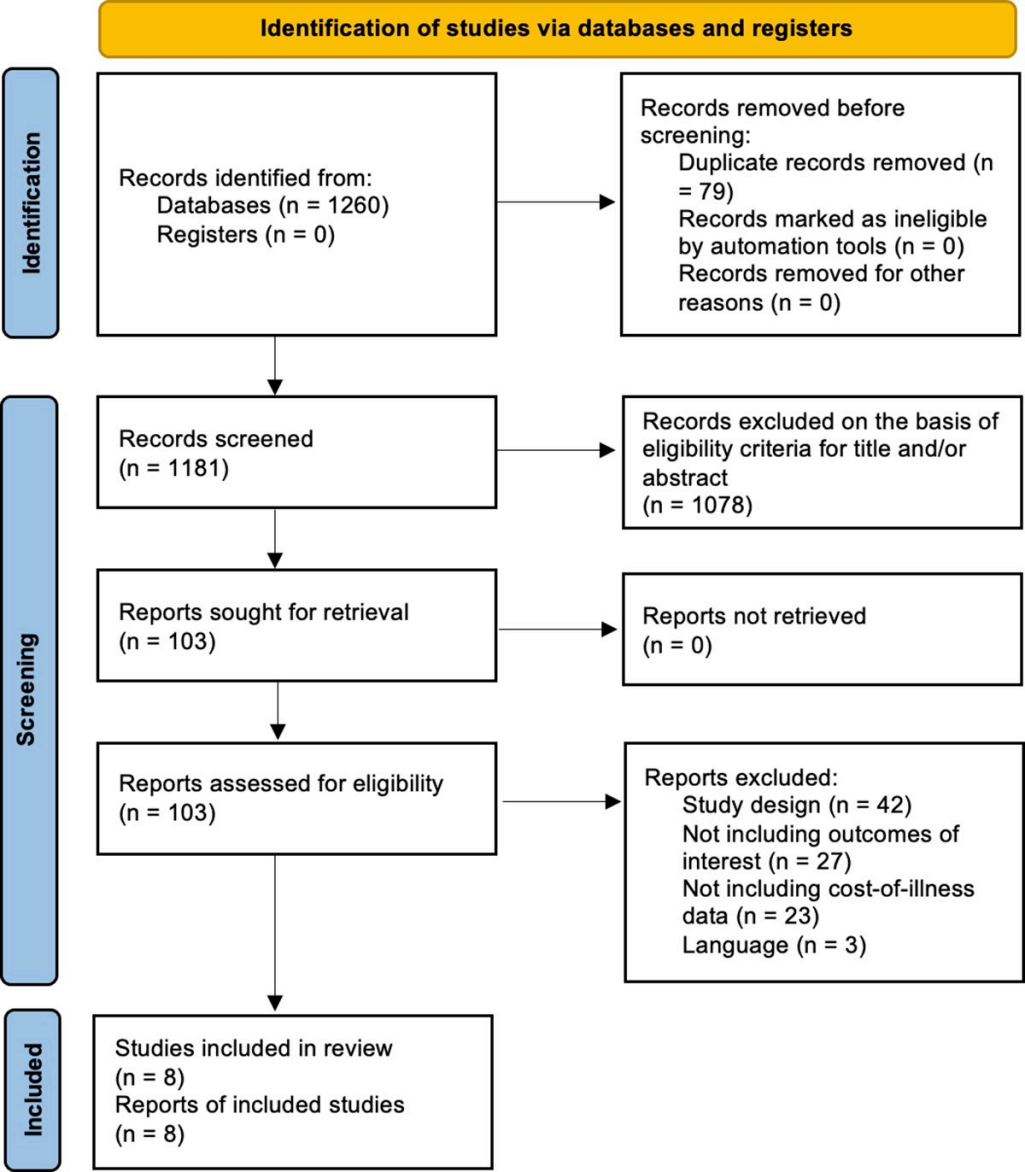
PRISMA 2020 flow chart of the included studies selection process.

The literature search yielded a total of 1260 studies. After reading the titles and abstract, 103 studies were selected for full reading and eight met the criteria for inclusion in the review (Fig 1) [4–6,11–15]. The main characteristics of each study included in the review are presented in S1 Table. Except for a research conducted in Turkey, all the included studies were published in the American continent, with the majority conducted in US (four studies), followed by one each for Brazil, Canada, and Mexico.

All the retrieved studies had an observational design and used a prevalence approach. The enrolled population and clinical outcomes considered in the economic analyses are listed in S2 Table (Supplementary material). Data on patients, clinical events and costs were sourced from hospital [6,11], national [4,12] and insurance [5,13–15] healthcare databases, with different level of details. In all reports, it was possible estimated the economic burden of CVD attributable to familial hypercholesterolemia, non-familial hypercholesterolemia, or mixed dyslipidemia, even if primary study objective and reported outcomes varied greatly across the reports (S1 and S2 Tables).

Year of costing ranged between 2002 and 2016 and the estimation cost approaches of the studies were heterogeneous. All of the eight included studies assessed direct costs for hospitalization. The inclusion of other direct costs varied among the studies: costs of drugs were included in four studies [4,12–14], outpatient care in four [4,5,14,15], emergency room accesses in three [5,13,14]. Regarding indirect costs, only one study considered productivity loss secondary to CVD in working-age population [6].

The included papers variously reported issues associated with the quality of the studies when evaluated with the CHEC checklist: all presented a moderate‐high quality, with some issues associated with the description of the study perspective and incremental analysis of costs and outcomes applied (S3 Table).

A complete overview with estimates in each selected study is detailed in Table 1. Three out of eight studies report cost estimates as annual average health expenditure on all population, ranging from $17 to $259 million [4,6,11]. One study sought to determine differences in medical care expenditures between familial hypercholesterolemia and non-familial hypercholesterolemia cohorts on the basis of revenue data from medical care service and estimated 11 years total revenues of $17,071 and $11,178 for the two cohorts respectively [15]. The remaining included studies annualized costs for patient experienced a CV event, calculating up to the third year after the event. In particular, Nichols et al. established a $17,848 per high-TG patient with statin-controlled cholesterol levels [14]. Fox et al. found that direct incremental costs ranged from $17,903 to $65,825 in the first year after the CV event [5], and most notably in the first 30 days following the event (acute period), driven primarily by inpatient costs for hospitalization and interventions [5,15].

**Table 1:** Cost estimates reported in each selected study.

*First author*, *Year*, *Country*	*Cost estimates*						
	*Direct costs*			*Direct cost items*	*Indirect costs*	*Indirect cost items*	*Total costs*
*Balbay*, 2019 [6], Turkey	From a total high-risk population of 1.9 million, FH group was estimated up to 205,557 adults. Annual estimation of direct health costs of $40 million for 2035. The study also reported a $265 million saving over 2015–2035 period, with lowering LDL-C intervention.			*NR*	Annual mean income lost due to CVD estimation of $83million for 2035. The study also reported a $427 million saving over 2015–2035 period, with lowering LDL-C intervention.	*NR*	Total costs will reach $123 million in 2035. A total $ 691 million saving could be supposed with lowering LDL-C intervention in 2015–2035 period
*Patel*, 2019 [15], US	In a cohort of 237 903 patients with hyperlipidemia (13.7% FH prevalence), comparison of annual revenue (*per* patient) from medical care service for years 2005–2015 between FH and non-FH:			*NR*	*NR*	*NR*	Total revenue (2005–2015) for FH $17,071 and non-FH $11,178
		*Non-FH*	*FH*				
	2005	$687	$810				
	2006	$724	$852				
	2007	$752	$902				
	2008	$790	$920				
	2009	$847	$983				
	2010	$874	$1,043				
	2011	$867	$1,044				
	2012	$907	$1,063				
	2013	$974	$1,166				
	2014	$1,093	$1,294				
	2015	$1,005	$1,307				
*Bahia*, 2018 [11], Brazil	Of the 245,981 CAD admissions/year in Brazil. annual mean costs for hospitalizations attributable to an underlying diagnosis of FH were estimated from $17,650,972 (with FH prevalence at 0.4%) to $31,448,466 (with FH prevalence 0.73%)			*NR*	*NR*	*NR*	Annual mean costs ranged from $17,650,972 to $31,448,466
*Besa-Creuz*, 2018 [4], Mexico	For all subjects >18 years old with a diagnosis of familial hypercholesterolemia, annual mean costs were estimated at $259,172.90			Annual mean costs of treatment $40,524.03; outpatient care $7,788.67; laboratory tests $2,455.46; drugs $2,453.66; hospitalizations $7,407.28; surgery $ 37,086.11; other complications and interventions $201,981.72	*NR*	*NR*	Annual mean costs $259,172.90
*Nichols*, 2018 [14], US	For 2,702 subjects aged 45 and older with ASCVD, costs *per* patient were:						
	High TG group—Mean annualized utilization per patient: $17,848 (95% CL, $17,224 to $18,473			Inpatient admissions for CVD $971; Total inpatient $4,459; Emergency room $1,153; Outpatient clinic $4,377; Hospital ambulatory $1,001; Day surgery $455; Dialysis $137; Pharmaceutical dispenses $6,053	*NR*	*NR*	High TG group—Mean annualized utilization per patient: $17,848 (95% CL, $17,224 to $18,473
	Normal TG group—Mean annualized utilization per patient: $16,884 (95% CL, $16,625 to $17,143)			Inpatient admissions for CVD $924; Total inpatient $4,287; Emergency room $1,083; Outpatient clinic $4,337; Hospital ambulatory $802; Day surgery $456; Dialysis $89; Pharmaceutical dispenses $5,868	*NR*	*NR*	Normal TG group—Mean annualized utilization per patient: $16,884 (95% CL, $16,625 to $17,143)
*Fox*, 2016 [5], US	From a total of 451,450 subjects included in the study, direct incremental costs *per* patient categorized by CV event type, ranging from $17,903 to $65,825 in the first year of follow-up; from $474 to $19,617 in the second, and $2,598 to $26,982 in the third.			Costs for the first year after CV event were estimated for subjects with history of CV event $41,168; modified CHD cohort $ 41,648; moderate-risk cohort $ 40,500, and low-risk cohort $ 39,869	*NR*	*NR*	In the first year of follow-up period post-CV event the direct incremental costs ranged from $17,903 to $65,825; from $474 to $19,617 in the second, and $2,598 to $26,982 in the third.
				Costs for the second year after CV event were estimated for subjects with history of CV event $9,436; modified CHD cohort $8,301; moderate-risk cohort $6,622, and low-risk cohort $5,900			
				Costs for the third year after CV event were estimated for subjects with history of CV event $11,400; modified CHD cohort $7,386; moderate-risk cohort $6,622, and low-risk cohort $4,704			
*Henk*, 2015 [13], US	In a total of 193,385 enrollees with hyperlipidemia, first year after CVE total costs *per* patient were: $41,937 (±72,513)			First year: Inpatient costs: $24,993; Emergency room costs: $586; Ambulatory costs $10,232 Office visit costs $2,237; Outpatient visit costs $7,995; Other medical costs $2,438; Pharmaceutical costs $3,689;	*NR*	*NR*	First year after CVE total costs: $41,937 (±72,513)
	Second year total costs: $16,786 (±48,132)			Second year: Inpatient costs: $5,727; Emergency room costs: $352; Ambulatory costs $7,413; Office visit costs $2,012; Outpatient visit costs $5.40; Other medical costs $1,367; Pharmaceutical costs $3,391			Second year total costs: $16,786 (±48,132)
	Third year total costs: $15,133 (±41,503)			Third year: Inpatient costs: $4,764; Emergency room costs: $290; Ambulatory costs $6,165; Office visit costs $1,740; Outpatient visit costs $3.94; Other medical costs $894; Pharmaceutical costs $3,645			Third year total costs: $15,133 (±41,503)
*Dragomir*, 2010 [12], Canada	In a cohort of 55,134 patients with hypercholesterolemia, for 26,585 low adherent patients, the total cost of hospitalization estimated for the follow-up period was $71.0 million, and the estimated total excess costs of hospitalization attributable to low adherence amounted to $9.5 million. For 28,549 high adherent patients, the total cost of hospitalization estimated for the follow-up period was $65.9 million, and the estimated total savings in costs of hospitalization attributable to high adherence amounted to $10.3 million			Predicted mean costs of hospitalization in hospitalized patients were $ 8,654 (8,358 to 8,944).	*NR*	*NR*	For 26,585 low adherent patients, the total cost of hospitalization estimated for the follow-up period was $71.0million, and the estimated total excess costs of hospitalization attributable to low adherence amounted to $9.5 million. For 28,549 high adherent patients, the total cost of hospitalization estimated for the follow-up period was $65.9 million, and the estimated total savings in costs of hospitalization attributable to high adherence amounted to $10.3 million


ASCVD, atherosclerotic cardiovascular disease; CAD, coronary artery disease; CVD, cardiovascular disease; CVE, cardiovascular event; LDL-C, low-density lipoprotein–cholesterol; HDL-C, high-density lipoprotein–cholesterol; TG, triglyceride; FH, familial hypercholesterolemia, NR, not reported.

According to Henk et al., in the first year after CV event patients reported a cost of $41,937 (±72,513), with the greater proportion of this expenditure due to myocardial infarction and revascularization procedures, while in the second and third year the mean cost decreased to $16,786 (±48,132) and $15,133 (±41,503) respectively, mainly related to ambulatory costs [13]. Dragomir et al. reported an average cost for hospitalization of $8,654 in hospitalized patients [12].

A few studies also explored costs comparing sub-population stratified on the basis of CV risk or treatment adherence. Nichols et al. highlighted a difference between the mean annualized costs for high TG patient and normal TG group ($17,848 vs. $16,884), both with background hypercholesterolemia [14]. Henk et al. described the total costs for the three years after a CV event, reporting a first-year cost of $44,093 for high-risk patient and $42,773 for low-risk; while reporting yearly costs of $8,905 for high-risk subject without CV event [11]. Lastly, in the Canadian study by Dragomir et al., the total cost of hospitalization due to CVD were higher in low adherent patients to anti-dyslipidemic treatment than in those who were more adherent ($71.0 million vs. $65.9 million) [12].

Table 2 presented the economic impact of hypercholesterolemia and mixed dyslipidemia adjusted for inflation rate, reporting the possible economic impact of these conditions in 2020.

**Table 2:** Presentation of costs for each study after transforming costs to US dollars using Purchasing Power Parities (PPP), by inflating 1% annually.

*First author*, *Year*, *Country*	*Year of costing*	*Currency*	*Total costs*: *direct + indirect*	PPP rate for the costing year *	*Total costs transformed to USD using PPP*	*Recalculated with 1% inflation rate*, *present value 2020 (USD)*
Bahia, 2018 [11], Brazil	2013	Brazilian Real	BRL 51,764,175	1.646	$ 31,448,466	$ 33,717,012
Balbay, 2019 [6], Turkey	2016	USD	-	-	$ 123,000,000 (2035)	-
Besa-Creuz, 2018 [4], Mexico	2016	Mexican Peso	MXN 2,188,974.31	8.446	$ 259,172.90	$ 269,696.36
Patel, 2019 [15], USA	2015	USD	$ 1,307 (*per* case)	-	-	$ 1,373.67
Fox, 2016 [5], USA	2012	USD	$ 46,890 (1^st^ year after)	-	-	$ 55,775.15
			$ 19,617 (2^nd^ year)			$ 21,242.40
Henk 2015 [13], USA	2012	USD	$ 20,621 (1^st^ year)	-	-	$ 22,329.59
			$ 16,786 (2^nd^ year)			$ 18,176.83
			$ 15,133 3^rd^ year)			$ 16,386,87
Nichols, 2018 [14], USA	2016	USD	$ 17,848	-	-	$ 18,572.70
Dragomir, 2010 [12], Canada	2002–2006	Canadian dollars	CAD 71,000,000	1.234 (2008)	$ 57,536,466.80	$ 66,136,684.90

*PPP*, Purchasing Power Parities; *USD*, US dollar; *CAD*, Canadian dollar.

## Discussion

This systematic review synthetized the available evidence relating the cost-of-illness studies conducted in patients with familial hypercholesterolemia, non-familial hypercholesterolemia or mixed dyslipidemia. The review describes the possible financial burden of these conditions for patients, healthcare systems and society, based on the current literature.

The first important finding was the significant scarcity of cost-of-illness studies on the economic impact of hypercholesterolemia, as well as the wide methodological heterogeneity among the few retrieved studies. Furthermore, there was a noteworthy absence of European studies meeting the inclusion criteria, constituting a significant lack of evidence for this region. As a whole, these aspects prevent from drawing firm conclusions on the current cost burden attributable to familial hypercholesterolemia, non-familial hypercholesterolemia or mixed dyslipidemia.

However, some points for future researches came from this systematic review. First, all the included studies highlighted that the health burden associated with incidence of CVD in hypercholesterolemic patients was significant, with an important economic burden in terms of healthcare resource consumption and direct and societal costs. Among the findings, the major costs were attributable to secondary prevention for patients who had experienced a previous CV event [4,5] and to those with familial hypercholesterolemia [4,6,11]. For the latter, the higher economic impact is mostly due to their life-time increased risk for CVD than that in non-familial hypercholesterolemia patients.

Among hyperlipidemic patients with new acute CV events, healthcare utilization and costs were significantly higher than for those without CV events, and remained higher after three years from the event [4]. In particular, the economic analyses conducted in US allowed to notice that the largest total costs occurred in the first year after the new CV event, and most notably in the first 30 days following the event (acute period), driven primarily by inpatient costs for hospitalization and interventions [5,15].

Almost none information about the economic impact of hypercholesterolemia and dyslipidemia was reported in literature. Only one study conducted in Turkey assessed the direct and indirect costs in familial hypercholesterolemia [6]. This was based on a simulation conducted with a burden model and reported that direct costs accounted for only one third of the overall economic burden. The indirect costs were responsible for the majority of the economic burden associated to familial hypercholesterolemia and relative CVD accounting for 67.5% of overall costs.

Although the economic burden due to these conditions cannot be standardized due to lack of comparability among the included studies, it should be stressed the quantification of overall health expenditure produced by CVDs, to better appreciate the cost impact attributable to the proportion of ASCVD. Indeed, costs per CV event were widely affected by type of analysis and cost-items considered, as well as, within the same context, largely vary in relation to the type of intervention required and the follow-up period. In USA, Henk et al. found that the average direct cost in the first 30 days following a CV event summed at $22,404. Similarly, Fox et al. estimated that, considering all CVEs, the direct health costs ranged from $17,903 to $65,825 in the first year of follow-up period [5]. As reported by Besa-Creuz and coll., in Mexico average annual costs for CV event can be fixed at $34,424, with estimated $8,583 for non-fatal acute myocardial infarction, $7,824.30 for acute stroke and up to $15,577 for post-acute CV intervention (e.g., revascularization) [4].

The clinical and economic impact of CV events is a well establish public health issue. Overall, CVDs pose a significant economic burden worldwide, reaching an estimation of more $863 billion with a rising trend [3]. CVD costs the United States about $219 billion each year [16]. In Europe, the estimated overall direct healthcare cost due to CVD was €111 billion a year [17], with country-based differences in cost estimation. For instance, in Italy, first-year-cost per CV event can reach €15,158 for non-fatal acute myocardial infarction, €8,081 for coronary revascularization, €9,975 for acute stroke [18–22]. Similarly, in Spain, those CV events were respectively valued at €6,876, €9,519, and €5,944 [23]. While, in Turkey the costs for CV event were estimated at US$3,411 for non-fatal acute myocardial infarction and $2,030 for acute cerebrovascular disease [6,24].

Briefly, the found evidence may serve as scoping review of the health burden associated with incidence of CVD in hypercholesterolemic and dyslipidemic patients, which was found to be relevant due to its important economic burden in terms of healthcare resource consumption and direct and societal costs. However, the majority of cost-of-illness studies focused on the economic impact associated to CV event in those patients without assessing the overall hypercholesterolemic population (with or without CVD) and without assessing subgroup with different severity. Indeed, only one study included in this review performed a cost-of-illness analysis of the compete hypercholesterolemic population (irrespectively of CVD), comparing the annual revenue of FH patients and non-FH per patient charged to the medical care service for years 2005–2015 [15].

Some limitations should be acknowledged while appreciating the findings of this study. Beyond the paucity of retrieved evidence, the differences in study design and variability in methods of calculation of the economic impact of the disease affected the comparability among them. The majority of the reports retrospectively used electronic databases that collected administrative data for general health purposes; thus, incompleteness of information could affect the reliability of data on hypercholesterolemic patients. Again, due to the nature of the databases sourced across the included studies, it was not possible to investigate possible differences in diagnostic criteria for hypercholesterolemia and mixed dyslipidemia, which may have affected the actual extent of these conditions. Many studies highlighted the lack of prevalence data on familial hypercholesterolemia, with a likely underestimation of its clinical and economic burden.

Despite these limitations, this review adds central points for future discussion on the knowledge of the economic impact of hypercholesterolemia and the costs of care associated to this condition and important insights to define new assessment of the burden of these conditions that are lacking. These data are the basic information required to guide healthcare decision makers in assessing the value of the available interventions and implementing the most valuables ones.

In conclusion, this study was intended to summarize evidence relating to the cost-of-illness studies conducted in patients with familial hypercholesterolemia, non-familial hypercholesterolemia or mixed dyslipidemia. Overall, this systematic review provides an overview of the scarcity of available evidence and the different methods used for the reporting of the economic burden of hypercholesterolemia and mixed dyslipidemia. However, the current information were presented to inform and guide decisions around the planning of stakeholder involvement within future research, with important implication for public health sector and novel therapies implementation.

## Supporting information

S1 Checklist PRISMA 2020 checklist. (DOCX)

S1 Table Study design and cost of illness methods reported in the selected study. (DOCX)

S2 Table Population characteristics and clinical outcomes reported in the selected study. (DOCX)

S3 Table CHEC-list–assessment of the quality of the studies. (DOCX)

S1 Appendix Search strategy. (DOCX)
