# Supplementary material for: The economic impact of hypercholesterolemia and mixed dyslipidemia: A systematic review of cost of illness studies
Source: PLoS One. 2021 Jul 12;16(7):e0254631. doi: 10.1371/journal.pone.0254631 (PMC8274865; doi:10.1371/journal.pone.0254631)
Supplement: S1 Table — (DOCX) [file pone.0254631.s002.docx]

**Table S1.** Study design and cost of illness methods reported in the selected study.

| *Reference* | *Methods* | | *Cost of illness methodology* | | | | *Estimation cost methods* |
| --- | --- | --- | --- | --- | --- | --- | --- |
| *First author, Year, Country* | *Study design and aim* | *Study populations* | *Data source* | *Year of data collection* | *Cost category and costs items included* | *Year and currency* |  |
| Bahia, 2018, Brazil | Cross selection study to estimate the economic impact of hospitalizations due to CAD attributable to FH in the Brazilian Unified Health Care System (SUS) | The study population comprised ≥ 20 years of age hospitalized patients with FH and primary diagnoses related to CAD: angina pectoris, acute myocardial infarction, other acute ischemic heart diseases, recurrent myocardial infarction, certain complications following acute myocardial infarction, and chronic ischemic heart disease | The abridged files of the Hospital Information System of the SUS (SIHSUS). | 2012  -  2014 | Direct costs:  1) hospitalization costs | Brazilian reais (R$) converted to international dollar (Intl$) through correction factor (1.646) defined by the World Bank for the year 2013 (Purchasing Power Parity, PPP) | Estimated the costs of CAD from the perspectives of the public and private sectors, including indirect costs of productivity loss. Through an analysis of the historical series of hospitalizations for acute myocardial infarction, angioplasty, and revascularization (1998-  2010) in the DATASUS database, the authors estimated the average costs per hospitalized patient to be R$ 5,236 and R$ 16,905 in the public and private health care systems, respectively. From the public perspective, the estimated values are higher than those shown in this study, possibly due to the inclusion of indirect costs attributed to loss of productivity associated with death or recovery time following the event until work activity is resumed. This share of costs accounted for 73% of the total costs. Using primary collected data, Ribeiro and cols. estimated the costs of CAD in a group of 147 patients attending a referral center in public and private health care systems in a state in southern Brazil. They demonstrated annual costs of outpatient treatment of R$ 1,488 in the public and R$ 2,094 in the private health care system for the year 2002 |

| *Reference* | *Methods* | | *Cost of illness methodology* | | | | *Estimation cost methods* |
| --- | --- | --- | --- | --- | --- | --- | --- |
| *First author, Year, Country* | *Study design and aim* | *Study populations* | *Data source* | *Year of data collection* | *Cost category and costs items included* | *Year and currency* |  |
| Balbay 2019,  Turkey | Simulation model based on cohort study  estimate the impact of addressing modifiable risk factors on the future burden of cardiovascular diseases (CVD) in the general population and in two high-risk populations (heterozygous familial hypercholesterolemia and secondary prevention) for Turkey | Global estimates of familial hypercholesterolemia  prevalence were applied to the cohort of  3.4 million adult population aged 20 years and older living with CVD | The average direct cost per IHD case reimbursed by the Social Security Institution was calculated from a sample dataset of 2,728 admitted cases (ICD I20–I25) to The University of Health Sciences Türkiye Yüksek İhtisas Hospital. The average direct cost of hospitalized cases of CeVD was obtained from the literature.  (DRG), with some adaptations | 2016 (January-March) | Direct  (hospitalization) and indirect costs:  (foregone income lost)  due to CVD | Turkish Lira converted to United States dollars (US$) using the Organization for Economic Co-Operation and Development (OECD) purchasing power parity (PPP) adjusted exchange rate for 2014 of 1.163. | Average direct cost per IHD case reimbursed and direct cost of hospitalized cases of CeVD was obtained from the literature. For indirect costs, the human capital approach was used, and leveraged existing data and estimates from the literature |

| *Reference* | *Methods* | | *Cost of illness methodology* | | | | *Estimation cost methods* |
| --- | --- | --- | --- | --- | --- | --- | --- |
| *First author, Year, Country* | *Study design and aim* | *Study populations* | *Data source* | *Year of data collection* | *Cost category and costs items included* | *Year and currency* |  |
| Besa-Creuz,  2018,  Mexico | Se desarrolla una evaluación económica del tipo análisis de costo de la enfermedad donde secuantifican los recursos médicos utilizados para el tratamiento de la hipercolesterolemia así como para sus complicaciones. El objetivo es Describir los costos y el impacto económico de la atención de pacientes diagnosticados con hipercolesterolemia en México en el año 2016 | Hipercolesterolemia Prevención primaria bajo y medianoriesgo de ECV. • Hipercolesterolemia Prevención primaria alto riesgo de ECV • Hipercolesterolemia Prevención secundaria con enfermedad coronaria del corazón (ECC)sin ECV. • Hipercolesterolemia Prevenciónsecundaria con enfermedad coronaria del corazón con ECV. • Hipercolesterolemia y Diabetes mellitus tipo 2. • Hipercolesterolemiafamiliar. | Los costos de los recursos médicos utilizados son obtenidos de los costos unitarios por nivel de atención del Instituto Mexicano del Seguro Social (IMSS)así como de las licitaciones publicadas e nel portal de compras del IMSS | 2016 | Direct Costs:  Costo promedio anual del tratamiento, Costos promedios de: consulta con especialista, pruebas de lab, tratamiento farm, Hospitalización, Cirugía, Procedimientos quirúrgicos, costo de complicaciones  e intervenciones  de la hipercolesterolemia | 2016, Pesos mexicanos | Se lleva a cabo un análisis de costo de la enfermedad en dos etapas con abordaje bottom-up “abajo hacia arriba”, identificando aquellos generadores de costo directamente relacionados con la atención médica, estimando el costo de cada componente por separado, para luego sumarlos y obtener el costo total. La primera etapa tiene como objetivo cuantificar todos los recursos médicos utilizados para el tratamiento de cada uno de los tipos de la HC y sus complicaciones, considerando los siguientes rubros: diagnóstico, tratamiento farmacológico, pruebas de laboratorio y gabinete, consulta médica con especialista y hospitalización; la segunda etapa consiste en determinar los precios unitarios correspondientes al recursos médicos. |

| *Reference* | *Methods* | | *Cost of illness methodology* | | | | *Estimation cost methods* |
| --- | --- | --- | --- | --- | --- | --- | --- |
| *First author, Year, Country* | *Study design and aim* | *Study populations* | *Data source* | *Year of data collection* | *Cost category and costs items included* | *Year and currency* |  |
| Patel,  2019,  USA | Retrospective observational study based on a  population-based cohort. The goal of this study is to use EHR-based algorithms to implement a population-based screening approach to identify the hidden burden of FH and study the trends of major adverse cardiovascular events (MACE), mortality and cost of care associated with this diagnosis. | Patients in the EHR database with ICD, Ninth Revision (ICD-9) codes for hyperlipidemia | EHR database - Geisinger Health System (GHS) | January 2000  to  August 2016 | N.R. | 2005-2015  US$ | Using a complete case analysis methodology, inflation adjusted median annual revenue for the FH cohort was higher for each year from 2005 through 2015. The total follow-up time for each patient during the study period for analysis (2005–2015) was used as an offset variable to normalize the total amount to a per-year basis. |

| *Reference* | *Methods* | | *Cost of illness methodology* | | | | *Estimation cost methods* |
| --- | --- | --- | --- | --- | --- | --- | --- |
| *First author, Year, Country* | *Study design and aim* | *Study populations* | *Data source* | *Year of data collection* | *Cost category and costs items included* | *Year and currency* |  |
| Dragomir, 2010, Canada | A cohort study to evaluate the risk of cardiovascular disease, hospitalization, and direct health-care costs related to low adherence to statins in the primary prevention of cardiovascular disease from the perspective of the health-care system | All patients who initiated statins therapy with atorvastatin, fluvastatin, lovastatin, pravastatin, simvastatin or rosuvastatin,  between January 1, 1999 and June 30, 2002, and who had not taken any statin in the 2 years before entry into the  cohort.  Patients had to be between 45 and 85 years of age and been covered for their drugs by the RAMQ for at least 2 years  before cohort entry.  The date of the first prescription for a statin agent was defined as the cohort entry date.  Furthermore, to be eligible, subjects had to be free of any diagnosis of cardiovascular disease as demonstrated by the absence of a vascular diagnosis or vascular medical procedure in  the 5 years before entering the cohort and no vascular drug  marker in the 2 years before the cohort entry date. | The data were collected from the Régie de l’Assurance Maladie du Québec (RAMQ) and Med-Echo databases, both of which administer public health-care insurance programs in the province of Québec, Canada. | 1999-2002, followed-up for a minimum of 3 years | Direct costs:: Medical costs, hospitalization costs (including nursing care, laboratory tests, drugs, laundry,  food, administration, and maintenance costs), drug related costs | Between 2002 2006 Canadian dollars ($) | All categories of costs were estimated based on all cause costs method and disease-related costs method. The all-cause costs method sums all the expenditure incurred in the period of analysis. disease-related costs method estimates costs by restricting its attention to expenditure (hospitalization, medical, and pharmaceutical) related to dyslipidemia or cardiovascular disease. |

| *Reference* | *Methods* | | *Cost of illness methodology* | | | | *Estimation cost methods* |
| --- | --- | --- | --- | --- | --- | --- | --- |
| *First author, Year, Country* | *Study design and aim* | *Study populations* | *Data source* | *Year of data collection* | *Cost category and costs items included* | *Year and currency* |  |
| Fox,  2016,  USA | Retrospective cohort study to evaluated the direct clinical and economic burden associated with new CVE up to 3 years post-event among patients with hyperlipidemia | Hyperlipidemic patients (age ≥18) with a primary inpatient claim for new CVE, patients were included in the study if they had ≥1 medical claims for hyperlipidemia (International Classification of Diseases, 9th Revision Clinical Modifications [ICD-9-CM] code 272 | IMS LifeLink PharMetrics Plus data | From January 1, 2006 through June 30, 2012 | Direct costs included inpatient, outpatient,  outpatient office, emergency room and pharmacy visits and  direct costs associated with healthcare utilization | 2012 US$ | Direct incremental costs (obtained from claims) incurred during the acute (first 1 month post-index date), and long-term (1, 2, 3 years post-index date) follow-up periods for hyperlipidemia patients, stratified by CVD risk level. cardiovascular event categorized by post event period: MI, IS, UA,PCI,CABG, HF, TIA |

| *Reference* | *Methods* | | *Cost of illness methodology* | | | | *Estimation cost methods* |
| --- | --- | --- | --- | --- | --- | --- | --- |
| *First author, Year, Country* | *Study design and aim* | *Study populations* | *Data source* | *Year of data collection* | *Cost category and costs items included* | *Year and currency* |  |
| Henk  2015,  USA | Retrospective cohort study to: 1. determine the mean costs associated with CVEs among patients with hyperlipidemia; 2. characterizing costs by CVE type and coronary heart disease (CHD) risk. | The study population comprised:  1. patients ≥18 years old with  hyperlipidemia with a CVE [myocardial  infarction (MI), ischemic stroke (IS), transient ischemic attack (TIA), heart failure (HF), unstable angina (UA), percutaneous coronary intervention (PCI), and coronary artery bypass  graft (CABG)] in commercial health plan enrollees only.  2. CVE risk level: Low risk: 0–1 CHD risk factors, Moderate risk: 2 CHD risk factors , High risk: any CHD or CHD risk equivalent.  3. Patients with no CVE. | The data were collected using longitudinal administrative healthcare claims data: Optum Research  Database (ORD), which contains de-identified  medical and pharmacy claims data annually for approximately 14 million individuals who are enrolled in a commercial (fully insured or self-insured employer line of business) or Medicare Advantage plan. | January 1, 2006 and July 31, 2012 | Direct costs:  Inpatient costs, Emergency room  costs, Ambulatory costs, Office visit costs, Outpatient visit costs, Pharmaceutical costs, Other medical costs. | 2012 US$ | Healthcare costs were calculated by  baseline level of CHD risk and type of CVE  during several follow-up periods: Acute (days 0–30), short-term (days 31–365), first year (days 0–365), second year (days 366–730), and third year (days 731–1095). Sum of all health plan- and patient-paid amounts for all medical (ambulatory [office and outpatient hospitalization] visits, emergency room visits, inpatient hospitalization, and other services) and retail pharmacy services during the time of enrollment in the health plan. |

| *Reference* | *Methods* | | *Cost of illness methodology* | | | | *Estimation cost methods* |
| --- | --- | --- | --- | --- | --- | --- | --- |
| *First author, Year, Country* | *Study design and aim* | *Study populations* | *Data source* | *Year of data collection* | *Cost category and costs items included* | *Year and currency* |  |
| Nichols, 2018  USA | Observational cohort study to Compare medical utilization and costs of patients with and without high TG (TRIGLYCERIDE) levels | Patients aged 45 and older with atherosclerotic cardiovascular disease (ASCVD) who had a TG level < 500 mg/dl in 2010, were receiving statin therapy but no other anti-hyperlipidemic agent, had LDL-C values between 40 and 100 mg/dl, and had a charted diagnosis of myocardial infarction, ischemic stroke, acute coronary syndrome, or peripheral artery disease. Excluded individuals with a life-threatening illness or charted heart failure diagnosis. Dividing high TG levels (200 to 499mg/dl) and normal TG levels (<150mg/dl). | EPIC based electronic health record (EHR) of Kaiser Permanente Northwest (KPNW) and Kaiser Permanente Southern California (KPSC) | 2010 and followed until death, disenrollment or the end of 2016 | Direct costs:  Patient admissions, emergency room visits, non-emergent ambulatory visits, and pharmaceutical dispenses per year of eligibility. . | 2016 US$ | Inpatient, outpatient, and pharmacy and total medical costs for each individual during their entire follow-up period. Costing methods were based on procedures developed and validated by the KPNW Center for Health Research. Cost models were adjusted for age, sex, race/ethnicity, study site, baseline costs, presence of diabetes, chronic kidney disease, obesity (BMI>30kg/m2), hypertension (BP>140/90mmHg), and low HDL cholesterol (<40mg/dl) to account for differences between groups that could impact follow-up costs.time of enrollment in the health plan. |

**References**

Balbay Y et al. The Impact of Addressing Modifiable Risk Factors to Reduce the Burden of Cardiovascular Disease in Turkey. Turk Kardiyol Dern Ars 2019;47(6):487-497.

Baeza-Cruz et al. Análisis de Costo dela Enfermedad, del Tratamiento, las Complicaciones e Intervenciones de la Hipercolesterolemia en México en 2016. Value in Health Regional Issues 2018;17:56–63.

Bahia LR et al. Estimated Costs of Hospitalization Due to Coronary Artery Disease Attributable to Familial Hypercholesterolemia in the Brazilian Public Health System. Arch Endocrinol Metab 2018;62(3):303-308.

Dragomir A et al. Relationship Between Adherence Level to Statins, Clinical Issues and Health-Care Costs in Real-Life Clinical Setting. Value Health. 2010;13(1):87-94.

Fox KM et al. Clinical and economic burden associated with cardiovascular events among patients with hyperlipidemia: a retrospective cohort study. BMC Cardiovasc Disord. 2016;16:13.

Henk HJ. A Retrospective Study to Examine Healthcare Costs Related to Cardiovascular Events in Individuals With Hyperlipidemia. Adv Ther 2015;32(11):1104-16

Nichols GA et al. Comparison of Medical Care Utilization and Costs Among Patients With Statin-Controlled Low-Density Lipoprotein Cholesterol With Versus Without Hypertriglyceridemia. Am J Cardiol 2018;122(7):1128-1132.

Patel P et al. Hidden Burden of Electronic Health Record-Identified Familial Hypercholesterolemia: Clinical Outcomes and Cost of Medical Care. J Am Heart Assoc. 2019;8(13):e011822.
