# Supplementary material for: The economic impact of hypercholesterolemia and mixed dyslipidemia: A systematic review of cost of illness studies
Source: PLoS One. 2021 Jul 12;16(7):e0254631. doi: 10.1371/journal.pone.0254631 (PMC8274865; doi:10.1371/journal.pone.0254631)
Supplement: S2 Table — (DOCX) [file pone.0254631.s003.docx]

**Table S2.** Population characteristics and clinical outcomes reported in the selected study

| *First author, Year, Country* | *Population demographic and clinical characteristics* | *Clinical outcomes* |
| --- | --- | --- |
| Bahia, 2018, Brazil | Assuming an international prevalence of FH of 0.4% and 0.73%, of the 245,981 CAD admissions/year in Brazil, approximately 7,249 and 12,915, respectively, would be attributable to an underlying diagnosis of FH. | Most hospitalizations (48%) occurred due to a diagnosis of angina (I20) in the age group between 45-74 years (76.3%) and in the Southeast region (47.8%). Men had more hospitalizations for all diagnosis of CAD than women (60.4% vs. 40.6%). ~~.~~ |
| Balbay, 2019, Turkey | Total high-risk population will reach 1.9 million in 2015. For FH, depending on the prevalence rate assumption (0.2%–0.5%), this group was estimated at between 82,223 and 205,557 adults in 2016 and is projected to increase to between 104,375 and 260,937 adults by 2035. | N.A. |
| Besa-Creuz, 2018, Mexico | N.R. | **Complicación y probabilidad anual:**  Evento cardiovascular 4.0%  Angina estable-episodio agudo 2.0%  Angina estable-episodio post-agudo 2.0%  Angina inestable-episodio agudo 2.0%  Angina inestable-episodio post-agudo 2.0%  Enfermedad coronaria grave 1.7%  Infarto al miocardio-episodio agudo 1.2%  Infarto al miocardio-episodio post-agudo 1.2%  Insuficiencia cardíaca-episodio agudo 1.2%  Insuficiencia cardíaca-episodio post-agudo 1.2%  Accidente cerebrovascular isquémico- episodio post-agudo 0.8%  Accidente cerebrovascular isquémico- episodio agudo 0.8%  Ataque isquémico transitorio-episodio agudo 0.8%  Ataque isquémico transitorio-episodio post- agudo 0.8%  **Intervenciones y Probabilidad:**  Revascularización 1.6%  Revascularización coronaria 1.6%  Intervención coronaria percutánea 0.3%  Bypass coronario 0.4% |
| Patel, 2019, USA | EHR FH was identified in 32 613 individuals, which was 2.7% of the 1.18 million EHR cohort and 13.7% of 237 903 patients with hyperlipidemia. | FH had higher rates of myocardial infarction (14.77% versus 8.33%; P<0.0001), heart failure (11.82% versus 10.50%; P<0.0001) |
| Dragomir, 2010, Canada | A cohort of 55,134 patients included were aged between 45 and 85,  initially free of cardiovascular disease, newly treated with statins. | The low adherence was associated with an increasing risk of coronary artery disease, cerebrovascular disease, and chronic heart failure by 7%, 13%, and 13%, respectively; and also with an increased rate of hospitalization of 4%. Moreover, by using a nested case control design, we previously reported that subjects who were high adherent (80%) were less likely to present a first coronary artery disease (RR: 0.82; 95% CI 0.77–0.87) compared to a level of <20%; high adherence (80%) to statin agents was also significantly decreased the risk of cerebrovascular disease by 22% (RR: 0.74; 0.65–0.84) compared with low adherence (<80%); and finally, high level of adherence to statins was associated with a 19% reduction of chronic heart failure  (RR:0.81; 0.71–0.91) |
| Fox, 2016, USA | A total of 451,450 patients were eligible for the study, among which 267,165 patients had a new CV event, and 184,285 patients had no new CV event before 1:1 matching | Among patients with a new CV event, a large proportion had two or more new CV events (65.8%) during the 3-year follow-up period. Myocardial infarction was the most common CVE in all the risk cohorts |
| Henk 2015, USA | 193,385 commercial enrollees with hyperlipidemia and a CVE.  Their average [± standard deviation (SD)] age was 62.0 (±12.1) years, male (62.5%), Low risk: 0–1 CHD risk factors 31,307 (16.19),  Moderate risk: 2 CHD risk factors 33,512 (17.33), High risk: any CHD or CHD risk equivalent 128,566 (66.48) | N.R. |
| Nichols, 2018, USA | 2,702 patients in the high TG group (age 66.8 years; men 62.8%; white 64.3%; BMI 31.2 g/m2; TG 257 mg/dl; LDL 75 mg/dl; HDL 41 mg/dl) and 14,481 in the normal TG group (age 69.5 years; men 64.2%; white 56.0%; BMI 28.7 g/m2; TG 98 mg/dl; LDL 76 mg/dl; HDL 51 mg/dl). | Normal TG group - Mean annualized utilization (95% confidence limits - 95%CL) per person: Inpatient admissions 0.23 (0.22 to 0.24); Inpatient days 0.91 (0.87 to 0.96); Emergency room visits 1.01 (0.99 to 1.03) |

**References**

Balbay Y et al. The Impact of Addressing Modifiable Risk Factors to Reduce the Burden of Cardiovascular Disease in Turkey. Turk Kardiyol Dern Ars 2019;47(6):487-497.

Baeza-Cruz et al. Análisis de Costo dela Enfermedad, del Tratamiento, las Complicaciones e Intervenciones de la Hipercolesterolemia en México en 2016. Value in Health Regional Issues 2018;17:56–63.

Bahia LR et al. Estimated Costs of Hospitalization Due to Coronary Artery Disease Attributable to Familial Hypercholesterolemia in the Brazilian Public Health System. Arch Endocrinol Metab 2018;62(3):303-308.

Dragomir A et al. Relationship Between Adherence Level to Statins, Clinical Issues and Health-Care Costs in Real-Life Clinical Setting. Value Health. 2010;13(1):87-94.

Fox KM et al. Clinical and economic burden associated with cardiovascular events among patients with hyperlipidemia: a retrospective cohort study. BMC Cardiovasc Disord. 2016;16:13.

Henk HJ. A Retrospective Study to Examine Healthcare Costs Related to Cardiovascular Events in Individuals With Hyperlipidemia. Adv Ther 2015;32(11):1104-16

Nichols GA et al. Comparison of Medical Care Utilization and Costs Among Patients With Statin-Controlled Low-Density Lipoprotein Cholesterol With Versus Without Hypertriglyceridemia. Am J Cardiol 2018;122(7):1128-1132.

Patel P et al. Hidden Burden of Electronic Health Record-Identified Familial Hypercholesterolemia: Clinical Outcomes and Cost of Medical Care. J Am Heart Assoc. 2019;8(13):e011822.
