# Supplementary material for: The economic impact of hypercholesterolemia and mixed dyslipidemia: A systematic review of cost of illness studies
Source: PLoS One. 2021 Jul 12;16(7):e0254631. doi: 10.1371/journal.pone.0254631 (PMC8274865; doi:10.1371/journal.pone.0254631)
Supplement: S3 Table — (DOCX) [file pone.0254631.s004.docx]

**Table S3.** CHEC-list – assessment of the quality of the studies (Evers et al., 2005).

| **Item** | Item No. | **Balbay 2019** | **Patel 2019** | **Bahiia 2018** | **Baeza-Cruz 2018** | **Nichols 2018** | **Fox 2016** | **Henk 2015** | **Dragomir 2010** |
| --- | --- | --- | --- | --- | --- | --- | --- | --- | --- |
| Is the study population clearly described? | 1 | ◘ | ◘ | ◘ | ◘ | ◘ | ◘ | ◘ | ◘ |
| Are competing alternatives clearly described? | 2 | ◘ | ◘ | ◘ | ◘ | ◘ | ◘ | ◘ | ◘ |
| Is a well-defined research question posed in answerable form? | 3 | ◘ | ◘ | ◘ | ◘ | ◘ | ◘ | ◘ | ◘ |
| Is the economic study design appropriate to the stated objective? | 4 | ◘ | ◘ | ◘ | ◘ | ◘ | ◘ | ◘ | ◘ |
| Is the chosen time horizon appropriate in order to include relevant costs and consequences? | 5 | ◘ | ◘ | ◘ | ◘ | ◘ | ◘ | ◘ | ◘ |
| Is the actual perspective chosen appropriate? | 6 | ◘ | ◘ | ◘ | ◘ | ◘ | ◘ | ◘ | ◘ |
| Are all important and relevant costs for each alternative identified? | 7 | ◘ | ◘ | ◘ | ◘ | ◘ | ◘ | ◘ | ◘ |
| Are all costs measured appropriately in physical units? | 8 | ◘ | ◘ | ◘ | ◘ | ◘ | ◘ | ◘ | ◘ |
| Are costs valued appropriately? | 9 | ◘ | ◘ | ◘ | ◘ | ◘ | ◘ | ◘ | ◘ |
| Are all important and relevant outcomes for each alternative identified? | 10 | ◘ | ◘ | ◘ | ◘ | ◘ | ◘ | ◘ | ◘ |
| Are all outcomes measured appropriately? | 11 | ◘ | ◘ | ◘ | ◘ | ◘ | ◘ | ◘ | ◘ |
| Are outcomes valued appropriately? | 12 | ◘ | ◘ | ◘ | ◘ | ◘ | ◘ | ◘ | ◘ |
| Is an incremental analysis of costs and outcomes of alternatives performed? | 13 | ◘ | ◘ | ◘ | ◘ | ◘ | ◘ | ◘ | ◘ |
| Are all future costs and outcomes discounted appropriately? | 14 | ◘ | ◘ | ◘ | ◘ | ◘ | ◘ | ◘ | ◘ |
| Are all important variables, whose values are uncertain, appropriately subjected to sensitivity analysis? | 15 | ◘ | ◘ | ◘ | ◘ | ◘ | ◘ | ◘ | ◘ |
| Do the conclusions follow from the data reported? | 16 | ◘ | ◘ | ◘ | ◘ | ◘ | ◘ | ◘ | ◘ |
| Does the study discuss the generalizability of the results to other settings and patient/client groups? | 17 | ◘ | ◘ | ◘ | ◘ | ◘ | ◘ | ◘ | ◘ |
| Does the article indicate that there is no potential conflict of interest of study researcher(s) and funder(s)? | 18 | ◘ | ◘ | ◘ | ◘ | ◘ | ◘ | ◘ | ◘ |
| Are ethical and distributional issues discussed appropriately? | 19 | ◘ | ◘ | ◘ | ◘ | ◘ | ◘ | ◘ | ◘ |
| ◘ Yes; ◘ No | | | | | | | | | |

Presentation of costs for each study after transforming costs to US dollars using Purchasing Power Parities (PPP), by inflating 1% annually

* For those studies that did not report the corresponding PPP rate, we gathered the PPPs and exchange rates. [Accessed 17-07-2020]; Available at: http://stats.oecd.org/Index.aspx?DataSetCode=SNA_Table4#.

**References**

Balbay Y et al. The Impact of Addressing Modifiable Risk Factors to Reduce the Burden of Cardiovascular Disease in Turkey. Turk Kardiyol Dern Ars 2019;47(6):487-497.

Baeza-Cruz et al. Análisis de Costo dela Enfermedad, del Tratamiento, las Complicaciones e Intervenciones de la Hipercolesterolemia en México en 2016. Value in Health Regional Issues 2018;17:56–63.

Bahia LR et al. Estimated Costs of Hospitalization Due to Coronary Artery Disease Attributable to Familial Hypercholesterolemia in the Brazilian Public Health System. Arch Endocrinol Metab 2018;62(3):303-308.

Dragomir A et al. Relationship Between Adherence Level to Statins, Clinical Issues and Health-Care Costs in Real-Life Clinical Setting. Value Health. 2010;13(1):87-94.

Evers S, Goossens M, de Vet H, van Tulder M, Ament A. Criteria list for assessment of methodological quality of economic evaluations: Consensus on Health Economic Criteria. Int J Technol Assess Health Care. Spring 2005;21(2):240-5.

Fox KM et al. Clinical and economic burden associated with cardiovascular events among patients with hyperlipidemia: a retrospective cohort study. BMC Cardiovasc Disord. 2016;16:13.

Henk HJ. A Retrospective Study to Examine Healthcare Costs Related to Cardiovascular Events in Individuals With Hyperlipidemia. Adv Ther 2015;32(11):1104-16

Nichols GA et al. Comparison of Medical Care Utilization and Costs Among Patients With Statin-Controlled Low-Density Lipoprotein Cholesterol With Versus Without Hypertriglyceridemia. Am J Cardiol 2018;122(7):1128-1132.

Patel P et al. Hidden Burden of Electronic Health Record-Identified Familial Hypercholesterolemia: Clinical Outcomes and Cost of Medical Care. J Am Heart Assoc. 2019;8(13):e011822.
