# Supplementary material for: The economic impact of hypercholesterolemia and mixed dyslipidemia: A systematic review of cost of illness studies
Source: PLoS One. 2021 Jul 12;16(7):e0254631. doi: 10.1371/journal.pone.0254631 (PMC8274865; doi:10.1371/journal.pone.0254631)
Supplement: S1 Appendix — (DOCX) [file pone.0254631.s005.docx]

**Appendix 1**

**Search Strategy**

"Hyperlipoproteinemia Type II/economics"[Mesh] OR "Dyslipidemias/economics"[Mesh] OR (("Hyperlipoproteinemia"[Mesh] OR “hypercholesterolaemia”[tiab] OR "Dyslipidemias"[Mesh] OR “mixed dyslipidaemia*”[tiab] OR “mixed dyslipidemia*”[tiab] OR dyslipidaemia*[tiab] OR dyslipidemia*[tiab]) AND ("Cost of Illness"[Mesh] OR “Costs and Cost Analysis”[Mesh] OR “costs of illness”[tiab] OR “cost analysis”[tiab] cost*[tiab] OR economic*[tiab] OR “economic burden”[tiab]))

*The search strategy was primarily used for PubMed and then adapted for the other electronic databases.*
